# Increased Eotaxin and MCP-1 Levels in Serum from Individuals with Periodontitis and in Human Gingival Fibroblasts Exposed to Pro-Inflammatory Cytokines

**DOI:** 10.1371/journal.pone.0134608

**Published:** 2015-08-04

**Authors:** Elisabeth A. Boström, Elin Kindstedt, Rima Sulniute, Py Palmqvist, Mirjam Majster, Cecilia Koskinen Holm, Stephanie Zwicker, Reuben Clark, Sebastian Önell, Ingegerd Johansson, Ulf H. Lerner, Pernilla Lundberg

**Affiliations:** 1 Karolinska Institutet, Division of Periodontology, Department of Dental Medicine, Stockholm, Sweden; 2 Umeå University, Department of Molecular Periodontology, Umeå, Sweden; 3 Umeå University, Department of Cariology, Umeå, Sweden; 4 University of Gothenburg, Sahlgrenska Academy, Centre for Bone and Arthritis Research, Gothenburg, Sweden; University of the Pacific, UNITED STATES

## Abstract

Periodontitis is a chronic inflammatory disease of tooth supporting tissues resulting in periodontal tissue destruction, which may ultimately lead to tooth loss. The disease is characterized by continuous leukocyte infiltration, likely mediated by local chemokine production but the pathogenic mechanisms are not fully elucidated. There are no reliable serologic biomarkers for the diagnosis of periodontitis, which is today based solely on the degree of local tissue destruction, and there is no available biological treatment tool. Prompted by the increasing interest in periodontitis and systemic inflammatory mediators we mapped serum cytokine and chemokine levels from periodontitis subjects and healthy controls. We used multivariate partial least squares (PLS) modeling and identified monocyte chemoattractant protein-1 (MCP-1) and eotaxin as clearly associated with periodontitis along with C-reactive protein (CRP), years of smoking and age, whereas the number of remaining teeth was associated with being healthy. Moreover, body mass index correlated significantly with serum MCP-1 and CRP, but not with eotaxin. We detected higher MCP-1 protein levels in inflamed gingival connective tissue compared to healthy but the eotaxin levels were undetectable. Primary human gingival fibroblasts displayed strongly increased expression of MCP-1 and eotaxin mRNA and protein when challenged with tumor necrosis factor-α (TNF-α and interleukin-1β (IL-1β), key mediators of periodontal inflammation. We also demonstrated that the upregulated chemokine expression was dependent on the NF-κΒ pathway. In summary, we identify higher levels of CRP, eotaxin and MCP-1 in serum of periodontitis patients. This, together with our finding that both CRP and MCP-1 correlates with BMI points towards an increased systemic inflammatory load in patients with periodontitis and high BMI. Targeting eotaxin and MCP-1 in periodontitis may result in reduced leukocyte infiltration and inflammation in periodontitis and maybe prevent tooth loss.

## Introduction

Periodontitis is characterized by loss of tooth supporting tissues driven by a local chronic inflammation. The clinical outcome may be tooth mobility or tooth loss, both disabling conditions for the patient. The fact that the severity of tissue destruction varies between individuals suggests that intrinsic differences in the host-response affect how the inflammatory process causes loss of tooth supporting tissues, including jawbone [1]. Increasing evidence suggests that periodontitis is reflected not only by an oral but also by a systemic increase in inflammatory mediators [2]. This may contribute to the reported relation to other inflammation associated conditions such as atherosclerosis [3], diabetes [4], increased body mass index (BMI) [5], and rheumatoid arthritis (RA) [6].

Periodontitis is an infectious disease caused by bacteria present in the biofilm on the tooth surfaces. The biofilm provides an ecological niche to microorganisms, which represents a wide array of antigenic challenges for the host response. Molecules released from the biofilm activate and trigger the inflammatory response, which includes migration of neutrophils, monocytes/macrophages, lymphocytes, and recruitment/activation of bone resorbing osteoclasts, leading to periodontal tissue destruction. Leukocytes, along with the resident cells in the periodontium, *e*.*g*. gingival- and periodontal ligament fibroblasts and vascular endothelial cells, do upon stimulation synthesize and secrete a broad spectrum of inflammatory mediators involved not only in host defense but also in tissue response [1]. The cellular- and molecular pathogenetic mechanisms of periodontitis are complex and still elusive which is displayed by the lack of established local or systemic biological markers.

It is known that cytokines, chemokines, arachidonic acid metabolites, and proteolytic enzymes by different mechanisms collectively contribute to periodontal soft tissue and jawbone destruction [1]. Certain cytokines are suggested to play a critical role in the pathogenesis of periodontitis including pro-inflammatory cytokines that enhance the inflammatory response, and anti-inflammatory cytokines that suppress the intensity of the cascade [7]. An imbalance between the two may be involved in the molecular pathogenesis of periodontitis. Pro-inflammatory interleukin-1β (IL-1β) and tumor necrosis factor-α (TNF-α) play a prominent role in periodontal inflammation and are elevated in gingival crevicular fluid (GCF) [8–11] and in gingival tissue in periodontitis [12,13]. The pivotal role of these cytokines in periodontitis is supported by reports that attachment loss is reduced in periodontitis patients with RA after anti-TNF treatment [14], and that local gingival administration of recombinant TNF-α or IL-1β exacerbates experimental periodontitis in rats [15,16]. These two cytokines are involved in the induction of several other inflammatory mediators in periodontal inflammation, such as IL-6, chemokines, matrix metalloproteinases (MMPs) and prostaglandin E2 (PGE_2_) [7].

Chemokines are inducible, chemotactic cytokines that by activation of specific chemokine receptors affect leukocyte migration through regulation of cytoskeletal rearrangement, integrin–dependent adhesion, as well as detachment of cells. Chemokines and their cognate receptors have been demonstrated to play an important role in several chronic inflammatory diseases, like atherosclerosis [17], RA [18,19], asthma [20,21], inflammatory bowel disease [22,23] and psoriasis [24].

To date, several reports have associated serum cytokines with periodontitis, however, there are no weighted analyses with different subject characteristics including serum cytokines and periodontitis parameters [25]. Therefore, we analyzed a spectrum of cytokines in serum from subjects with and without periodontitis and evaluated the relative importance of each independent variable to the periodontal status by multivariate partial least squares (PLS) regression analyses. Next, we investigated if the source of the periodontitis-associated serum cytokines was inflamed gingiva and we analyzed if gingival fibroblasts, the most abundant cell type in the periodontium, express the cytokines and by which signaling pathway pro-inflammatory cytokines regulated their expression.

## Materials and Methods

### Study group

Adults, over 35 years of age, with periodontitis but no other known disease, who entered the Specialist clinic for periodontology at Norrlands University hospital, Umeå, Sweden between 2009 and 2012 were eligible for inclusion. An experienced specialist in periodontology examined all patients. The inclusion criteria were: (*i*) having moderate-severe periodontal injuries with at least 50% of the teeth showing bone loss exceeding more than 1/3 of the root length, bleeding on probing (BOP) at more than 20% of the pockets, and having more than 15 own teeth. The exclusion criteria were: (*i*) use of antibiotics or periodontal treatment in the previous 3 months, (*ii*) pregnancy or lactancy, or (*iii*) having any general disease or ongoing therapy with any anti-inflammatory drug. A periodontally healthy group was recruited at the Public dental health clinic at Norrlands University hospital, Umeå, Sweden. The inclusion criteria for the healthy subjects were: (*i*) having no signs of periodontal attachment loss and ≥24 teeth, with <3 mm distance between the cement-enamel junction and the alveolar bone margin, and a probing pocket depth (PPD) <4 mm; and (*ii*) being ≥35 years. The exclusion criteria were the same as for the periodontitis group.

Three additional periodontitis patients who met the inclusion criteria and underwent periodontal surgery for infection control was included in the study for tissue chemokine analyses. During flap surgery gingival tissue from inflamed periodontitis sites (BOP, PPD >6 mm and bone loss exceeding more than 1/3 of the root length) was collected. Moreover, the flap was extended to periodontally healthy sites in the same patient (no BOP, PPD <4 mm and <3 mm distance between the cement-enamel junction and the alveolar bone margin) and a gingival biopsy was collected. A tissue piece of approximately 0.5 x 0.5 x 0.5 cm was excised and the distance between the inflamed and non-inflamed site was approximately two centimetres. The tissue samples were placed on ice and analysed by ELISA (see below).

The Regional Ethical Review Board in Umeå, Sweden approved the study and written consent was received from all participants.

### Clinical Data Collection

The periodontal examination included periodontal probing and x-ray documentation. The parameters recorded were: number of teeth, BOP, and PPD over 4 mm (PPD ≥4 mm).

Information on medical status, education and lifestyle variables were obtained by interviews, body weight (kg) and height (cm) were measured when subjects wore light clothes but no shoes. Body mass index (BMI) was calculated as body weight (kg) divided by height squared (m^2^).

### Blood sampling

A venous blood sample of 10 ml was collected from each participant into a heparinized tube at inclusion. The participants were not fasted at the time of the blood sampling. Collection and handling of blood samples, including fractionation into plasma, serum and buffy coat, and storage at -80°C followed the standardized routines at Medical Biobank of Northern Sweden, Västerbotten County Council, Sweden.

### Measurements of Inflammatory mediators

Measurements of a panel of cytokines and chemokines in serum were performed using Luminex technology on a Bioplex Suspension Array System (Bio-Rad Laboratories Inc, Hercules, CA, USA) with a Milliplex Map kit (Millipore, Billerica, MA, USA). C- reactive protein (CRP) levels were determined using high sensitive immunoturbidimetric assay (CRPL3, Cobas C system, Roche Diagnostics, IN, USA).

### Fibroblast cultures

Gingival fibroblasts were isolated from gingival papillar explants obtained from four periodontally and systemically (no medication or general disease) healthy donors having no signs of periodontal attachment loss, <3 mm distance between the cement-enamel junction and the alveolar bone margin, and a probing pocket depth (PPD) <4 mm, and no bleeding on probing. Their rights were protected by the Regional Ethical Review Board at Umeå University, Umeå, Sweden. Verbal information was given and written consents were received.

Gingival explants were placed at the bottom of culture dishes 60 cm^2^ (Nunc, Roskilde, Denmark) with α modification of Minimum Essential Medium (α-MEM) supplemented with 10% foetal calf serum (FCS, Gibco-Brl/Life Technologies, Paisley, UK), L-glutamine (Gibco-Brl/Life technologies, Paisley, UK) and antibiotics (Meda AB, Solna, Sweden and Sigma-Aldrich, St. Louis, MO, USA), referred to as basic medium, and left untouched for 7–10 days until outgrowth of fibroblasts from the explants was observed. The fibroblasts were then detached and seeded in 24-well plates at a density of 5x10^4^/cm^2^. After attachment overnight, basic medium was changed and cells were incubated in the absence (control group) or presence of the test substances TNF-α or IL-1β (R&D Systems, Inc, Minneapolis, MN, USA) or the pharmacological inhibitors BMS-345541 (cat. no. B9935, Sigma Aldrich, St. Louis, MO, USA), Celastrol (cat. no. CO869, Sigma Aldrich, St. Louis, MO, USA and IKK-2 inhibitor V (cat. no. 401482, Millipore, Billerica, MA, USA) for different time periods, as indicated in the figure legends. Cells used in the experiments demonstrated a fibroblastic morphology and were used at passages 5–10. Cell culture supernatants were saved for analysis of eotaxin and MCP-1, and cell lysates were subjected to RNA-isolation. All experiments involving human gingival fibroblasts were repeated at least twice with similar results. Means for control and test groups are based on a number of 4 wells/group.

### Enzyme-linked Immunosorbent Assay (ELISA)

Gingival tissue samples were homogenized in ice-cold lysis buffer containing 20 mM HEPES (pH 7.4), 150 mM NaCl, 1 mM EGTA, 1% Nonidet P-40, 1% of proteinase inhibitor mixture (Sigma Aldrich, St. Louis, MO, USA), 0.5 mM pervanadate (Sigma Aldrich, St. Louis, MO, USA) and 1 mM phenylmethylsulfonyl fluoride (Sigma Aldrich, St. Louis, MO, USA). Total protein concentrations in tissue lysates were quantified using the Pierce BCA protein assay (Pierce Biotechnology, Rockford, IL, USA) and the lysates were diluted to obtain an equal total protein concentration of 1 mg/ml. Eotaxin and MCP-1 protein levels were measured in single samples of cell culture supernatants and in gingival tissue lysates with ELISA kits (cat. no DTX00 & DCP00, R&D Systems Inc, Minneapolis, MN, USA). The minimum detectable dose of the kits were 5 ng/ml for eotaxin and 1,7 ng/ml for MCP-1. Readings were made at 450 nm with a microplate spectrophotometer (SpectraMAX 340, Molecular Devices, Sunnyvale, CA, USA).

### RNA isolation and first-strand cDNA synthesis

Total RNA from gingival fibroblast cell cultures was isolated and DNAse treated using the RNAqueous–4PCR kit or RNAqueous Micro Kit (Ambion, Austin, TX, USA). Both kits were used according to instructions provided by the manufacturer. High Capacity cDNA Reverse Transcription Kit (Applied Biosystems, Foster City, CA, USA) was used to transcribe mRNA to cDNA.

### Quantitative real-time polymerase chain reaction (qPCR)

Taq-man (ABI PRISM 7900HT Sequence Detection System) was used to detect and analyze gene expression. The mRNA levels of *CCL11* (encoding eotaxin-1) and *CCL2* (encoding MCP-1) were determined using specific primers/fluorescent probe mix. Assay ID:

MCP-1 Hs00234140, eotaxin; custom made, 768804, hRPL13a; custom made, 773682 (Applied Biosystems, Foster City, CA, USA). To rule out the possibility of DNA contamination, samples in which the reverse transcription reaction had been omitted were also submitted to the PCR reaction, yielding no amplification. To control variability in amplification, *h-RPL-13a* was used as a housekeeping gene. All samples were run in duplicates. The relative expression of target mRNA was computed from the target Ct values and *h-RPL-13a* Ct values using the standard curve method (*User Bullentin #2*, Applied Biosystems).

### Western blot

Human gingival fibroblasts, isolated as previously described, were seeded in 12-well culture plates (2×10^5^ cells/well) and incubated for 5, 10, 15 and 30 min with or without IL-1β (100 pg/ml) or TNF-α (50 ng/ml) (R&D systems, Inc, Minneapolis, USA). Cells were collected, lysed and protein concentration was measured as described above (ELISA section). The lysates were resolved by 10% TRIS-HCl polyacrylamide gel electrophoresis and proteins were transferred onto a nitrocellulose membrane. Protein detection was performed using primary polyclonal rabbit anti-human Iκα antibodies (clone C-21, cat. no. sc-371, Santa Cruz Biotechnology, CA, USA) or monoclonal anti-β-actin (cat. no. A5441, Sigma Aldrich, St. Louis, MO, USA) followed by secondary horseradish peroxidase-conjugated anti-rabbit (cat. no. P0448, Dako, Glostrup, Denmark) and anti-mouse (cat. no. 31340, Thermo Scientific, Rockford, IL, USA) antibodies respectively. The immunoreactive proteins were visualized using the ECL Plus Western Blotting Detection System (GE Healthcare, Uppsala, Sweden) and analyzed by ImageQuant LAS 4000 imager.

### Data handling and statistical analysis

#### In vivo data

Dichotomized categorical data, *i*.*e*. sex, level of education (university/college education or not), smoking status (present or past smoking versus never smoking), use of Swedish snuff (present use versus no use), and having detectable levels of an inflammatory marker or not are presented as proportions (per cent), and differences in distribution between periodontitis and periodontally healthy tested with a Chi^2^ test. For continuous, normally distributed variables (variables in Table 1 and concentrations of eotaxin, MCP-1) means with 95% confidence interval (CI) are presented, and for log transformed variables (CRP) geometric means with 95% CI. Means were standardized for potential confounders as described in footnotes in Table 1 using the general linear model (GLM) procedure. Differences between group means for periodontitis cases and periodontally healthy controls were tested with Student´s *t*-test. In univariate analyses, variables significantly associated with periodontitis in the multivariate model were tested one-sided. With a cohort of 84 subjects, a moderate effect size (0.5), and an α-value of 0.05, we have 95% power to correctly reject H_0_ when it is false. Pearson correlation coefficients were calculated between serum concentrations of the inflammatory markers, BMI, and markers for periodontal status with logarithmic transformation of non-normally distributed variables. P-values <0.05 were considered statistically significant. For these analyses SPSS (version 22.0; IBM Corporation, Armonk, NY, USA) was used.

**Table 1:** Characteristics of study participants describing periodontitis (PD) and periodontally healthy (PH) subjects.

	PD (n = 43)	PH (n = 41)	p-value
Sex, % men/women^a^	53/47	39/61	0.184
Age in years, mean (95% CI)^b^	55.3 (51.9–58.7)	44.6 (42.3–46.9)	<0.001
BMI, mean, (95% CI)^b^ ^,^ ^c^	27.9 (26.5–29.4)	25.7 (24.3–27.2)	0.055
Education, % college/university^a^	32.6	68.3	<0.001
Snuff, % user^a^	18.6	24.4	0.518
Number of years as smoker, mean (95% CI)^b^ ^,^ ^c^	19.6 (14.8–24.3)	6.3 (1.4–11.3)	0.001
Proportion never-smoker, %^a^	27.9	75.6	<0.001
Number of teeth, mean (95% CI)^b^ ^,^ ^c^	24.9 (24.1–25.7)	27.0 (26.2–27.8)	0.001
BVS, %^b^ ^,^ ^c^	38.8 (33.7–44.0)	8.9 (3.6–14.2)	<0.001
Number of teeth with pocket ≥4 mm, mean (95% CI)^b^ ^,^ ^d^	20.8 (19.6–22.1)	0	<0.001
Number of teeth with bone loss ≥1/3 of the root length, mean (95% CI)^b^ ^,^ ^d^	19.3 (17.7–20.8)	0	<0.001

a) Distribution of numbers was tested with Chi^2^ test.

b) Differences between means were tested with Student´s *t*-test.

c) Standardized for sex, age and education using general linear modeling.

d) Standardized for sex, age, education and number of teeth using general linear modeling.

Partial least squares (PLS) modeling was used to detect correlations between subject characteristics and inflammatory markers having periodontitis or not as dependent variable. The software SIMCA P+ (v. 12.0; Umetrics AB, Umeå, Sweden) was used. The independent block was formed by plasma concentrations of all measured inflammatory markers, age, gender, education, BMI, number of years of smoking, use of snuff, blood pressure and number of teeth. All variables were auto scaled to unit variance and, except for eotaxin and MCP-1, the inflammatory marker concentrations were logarithmically transformed before entered into the model. Further, principal component analysis (PCA) was applied to evaluate clustering by smoking status and inflammatory markers including never and present smokers only. The importance of each independent variable in explaining the variation among the outcome variable (periodontal status) is given in a PLS loading column plot with PLS correlation coefficients and 95% CI. The R^2^ and Q^2^ values give the capacity of the x-variables to explain (R^2^) and predict (Q^2^) the variance for the y-value (periodontal status). Q^2^ values were obtained by cross-validation where every 7th observation was kept out of the model and predicted by a model from the remaining observations. This was repeated until all observations had been kept out once.

#### In vitro data

The statistical analyses were performed using one-way analysis of variance (ANOVA) with Levene’s Homogeneity test, and post-hoc Bonferroni’s, Tukeys or where appropriate Dunnett’s test. All experiments were performed at least twice with comparable results. Data are presented as means ± standard error of means (SEM). The significance levels were set to *P* < 0.05 (*), 0.01 (**) or 0.001 (***).

## Results

### Increased serum levels of the inflammatory markers CRP, eotaxin and MCP-1 in subjects with periodontitis

Characteristics of 43 patients with periodontitis and 41 periodontally healthy subjects, all without any general disease, are presented in Table 1. There was no difference in the proportion of men and women between the groups, but periodontitis patients were approximately 10 years older than the healthy subjects. Furthermore, the number of teeth was lower, the proportion of smokers higher, the education level lower, and BMI tended to be higher in the periodontitis group, whereas use of Swedish snuff (a powder tobacco product) did not differ between the groups. In accordance with the inclusion criteria, periodontitis patients had a significantly higher proportion of surfaces with BOP, number of teeth with a periodontal pocket equal to or deeper than 4 mm, and significantly higher number of teeth with a bone loss exceeding one third of the root length.

We analyzed CRP and a spectrum of inflammatory markers in serum from periodontitis and healthy subjects: eotaxin, MCP-1, CRP, IL-1β IL-4, IL-6, IL-10, IL-12, IL-13, IL-17, TNF-α IFN-γ, fibroblast growth factor 2 (FGF2), macrophage inflammatory protein 1 alpha (MIP-1α), macrophage derived chemokine (MDC) and assessed their correlations to patient characteristics. Serum levels of CRP correlated significantly with the numbers of bleeding pockets (r_Pearson_ = 0.353, *P* = 0.001), number of teeth with pockets ≥4 mm (r_Pearson_ = 0.355, *P* = 0.001), and bone loss (r_Pearson_ = 0.274, *P* = 0.012). No correlation was seen between these outcomes and any of the other analyzed cytokines (data not shown). BMI correlated significantly with MCP-1 (r_Pearson_ = 0.331, *P* = 0.003), and CRP (r_Pearson_ = 0.453, *P*<0.001), but not with eotaxin (r_Pearson_ = -0.171, *P* = 0.130) (Table 2). This correlation pattern was consistent among periodontitis patients and healthy subjects. Eotaxin and MCP-1 levels correlated significantly to each other (r_Pearson_ = 0.479, *P* = 0.002) in periodontitis patients but not healthy subjects (Table 2). As the distribution between groups regarding age and smoking was skewed we also assessed the correlations between eotaxin or MCP-1 and smoking and age, respectively, however we found no significant correlations (data not shown).

**Table 2:** Pearson correlation coefficients between serum CRP, MCP-1, eotaxin, and BMI in periodontitis (PD) and in periodontally healthy (PH) subjects.

	BMI		CRP (mg/l)1		Eotaxin (pg/ml)		MCP-1 (pg/ml)	
	Corr coeff	p-value	Corr coeff	p-value	Corr coeff	p-value	Corr coeff	p-value
CRP (mg/l)^1^								
All	0.453	<0.001	-	-	-0.232	0.038	0.223	0.047
PD	0.421	0.005	-	-	-0.343	0.031	0.204	0.207
PH	0.488	0.001			-0.266	0.097	0.180	0.266
Eotaxin (pg/ml)								
All	-0.171	0.130	-0.232	0.038	-	-	0.417	<0.001
PD	-0.130	0.422	-0.343	0.031	-	-	0.479	0.002
PH	-0.246	0.126	-0.266	0.097	-	-	0.286	0.073
MCP-1 (pg/ml)								
All	0.331	0.003	0.223	0.047	0.417	<0.001	-	-
PD	0.301	0.059	0.204	0.207	0.479	0.002	-	-
PH	0.373	0.018	0.180	0.266	0.286	0.073	-	-

1) Log10 values

**Fig 1:**
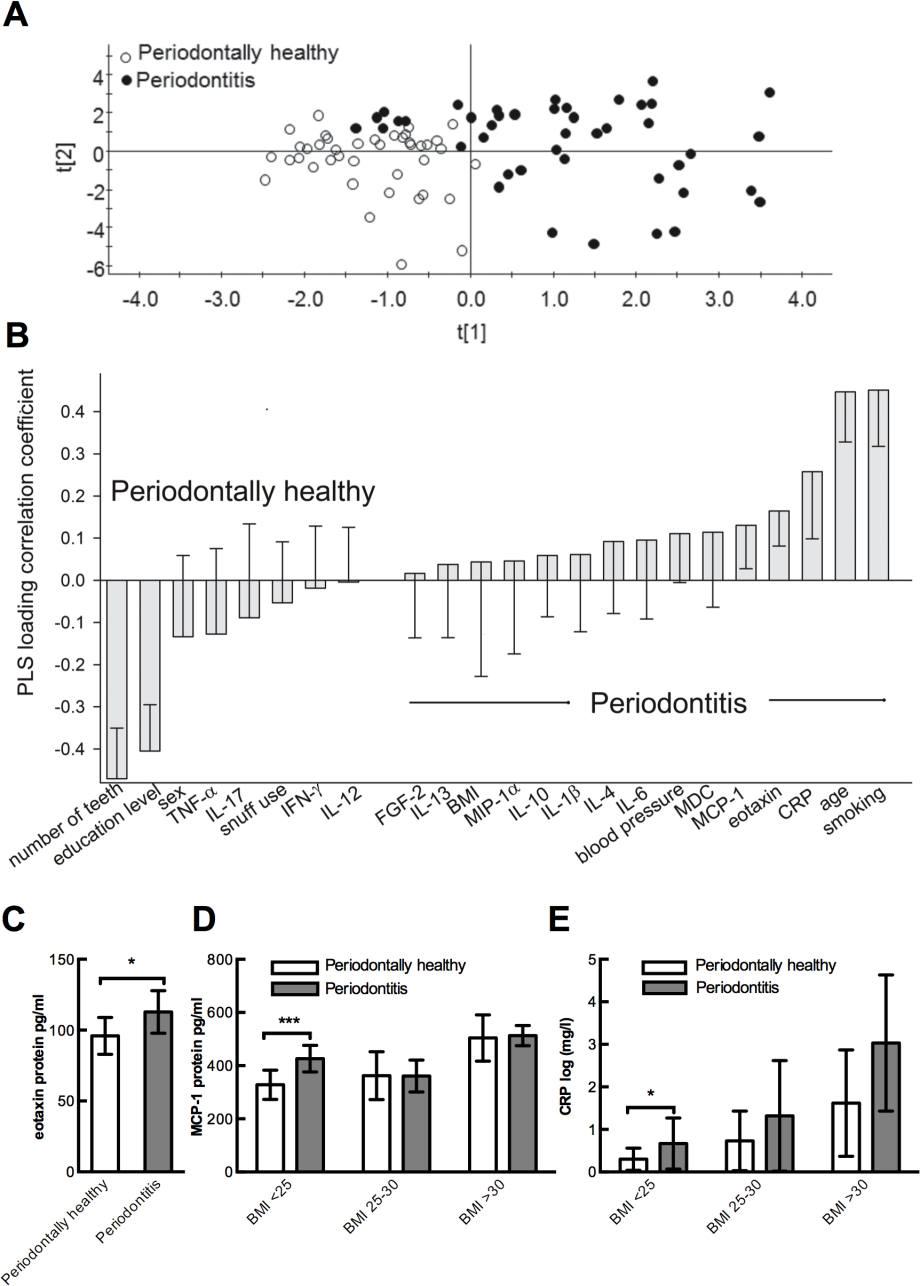
Multivariate PLS modeling results in a model with clustered periodontitis cases from periodontally healthy and significantly enhanced levels of inflammatory mediators in serum from periodontally diseased. **(A)** PLS scatter plot illustrating separation of periodontally diseased from healthy subjects by their serum concentrations of inflammatory markers and subject characteristics (see labels in B). The scores t1 and t2 are the new PCS created variables summarizing the x variables; **(B)** PLS column loading plot showing PLS correlation coefficients with 95% CI for the variables in the model, *i*.*e*. the model behind the separation in A. Bars for which the 95% whisker does not pass zero are statistically significant. Serum levels of eotaxin (pg/ml) **(C)**, MCP-1 (pg/ml) **(D)**, and CRP (mg/l) **(E)** in individuals with periodontitis and in periodontally healthy. Data are presented as arithmetic means (eotaxin and MCP-1) or geometric mean (CRP) with 95% confidence intervals. To circumvent confounding by BMI, data for MCP-1 and CRP are presented in BMI strata, i.e. BMI<25, BMI 25–30, BMI>30.

Multivariate PLS modeling employing serum concentration of all analyzed inflammatory markers, and a set of subject characteristics resulted in a model, which clustered periodontitis patients from periodontally healthy subjects (Fig 1A). The explanatory power (R^2^) was 54.4% and the cross validated predictive power (Q^2^) 44.3%. Concentrations of CRP, eotaxin, and MCP-1, in addition to years of smoking and age, were significantly associated with periodontitis, whereas more own teeth and high education were associated with being periodontally healthy (Fig 1B). No clustering of present versus never smokers was found by PCA employing the serum cytokines and chemokines (S1 Fig). Exclusion of outliers did not alter the result.

Following the positive correlation between BMI and serum CRP and MCP-1 levels, respectively, group comparisons for these parameters were performed in BMI strata (normal weight (BMI≤ 25), overweight (BMI >25 − <30) and obese (BMI≥30)). For eotaxin, which was unrelated to BMI, merged group means were compared. Eotaxin values (mean (95% CI)) were significantly higher in the periodontitis group than in the healthy group 113 (99–127) versus 96 (82–110) pg/ml, *P* = 0.049, (Fig 1C). Normal weight subjects with periodontitis had, compared to normal weight healthy subjects, higher levels of MCP-1 (pg/ml; mean (95% CI): 426 (376–476) versus 328 (274–382), *P* = 0.006, Fig 1D) and CRP (mg/l; mean (95% CI): 0.67 (0.39–2.84) versus 0.30 (0.17–0.51) mg/l, *P* = 0.019, Fig 1E). There was no difference in MCP-1 or CRP levels among overweight or obese subjects (Fig 1C and 1D). In accordance with the results from the multivariate model, the levels of IL-1β IL-4, IL-6, IL-10, IL-12, IL-13, IL-17, TNF-αIFN-γ, FGF-2, macrophage inflammatory protein 1 alpha (MIP-1α) and macrophage-derived chemokine (MDC) did not differ between patients with periodontitis and healthy subjects (S1 Table).

### Presence of eotaxin and MCP-1 in inflamed gingival connective tissue

**Fig 2:**
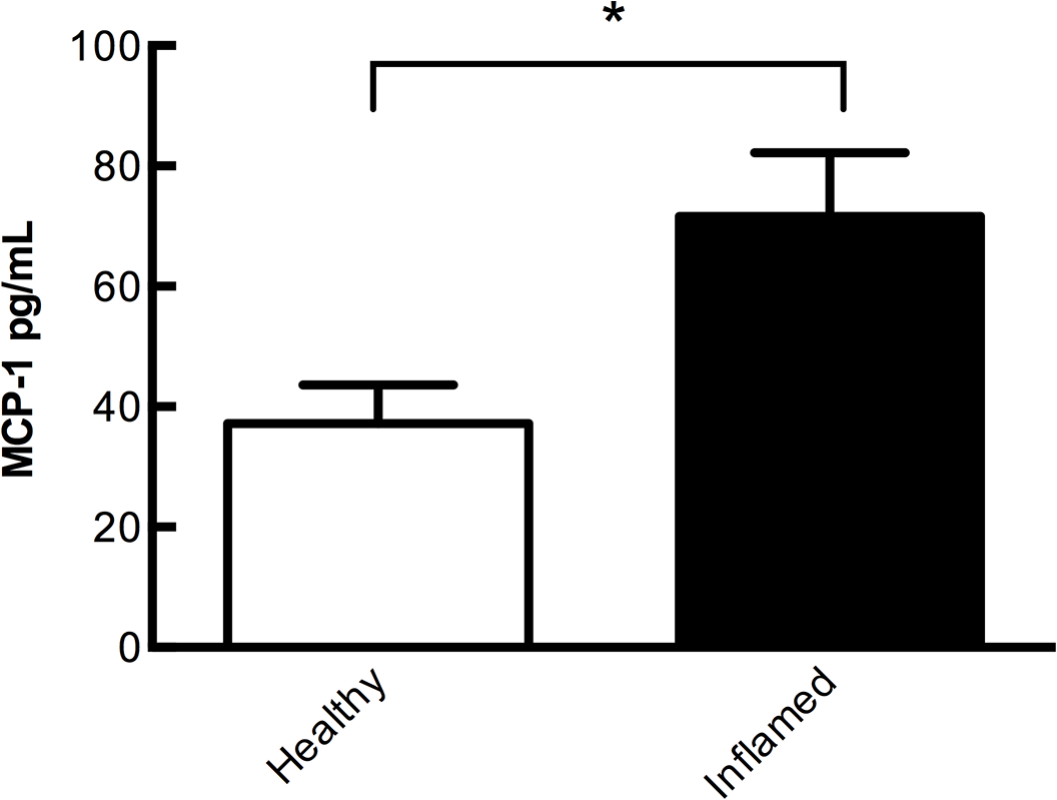
Presence of MCP-1 in inflamed gingival connective tissue. MCP-1 protein concentration measured by ELISA in gingival tissue homogenates of periodontitis (n = 3) and periodontally healthy (n = 3) subjects. Data expressed as means ± SEM.

We quantitatively assessed protein levels of MCP-1 in healthy and periodontitis gingival connective tissue homogenates with 2-fold, significantly, higher levels in inflamed periodontitis connective tissues (72 pg/ml) compared to healthy (37 pg/ml), *P*<0.05 (Fig 2). The eotaxin levels in connective tissue homogenates were undetectable in both healthy and inflamed periodontitis gingival tissue (data not shown).

### Time- and concentration dependent regulation of eotaxin and MCP-1 protein expression by TNF-α and IL-1β in primary human gingival fibroblasts

**Fig 3:**
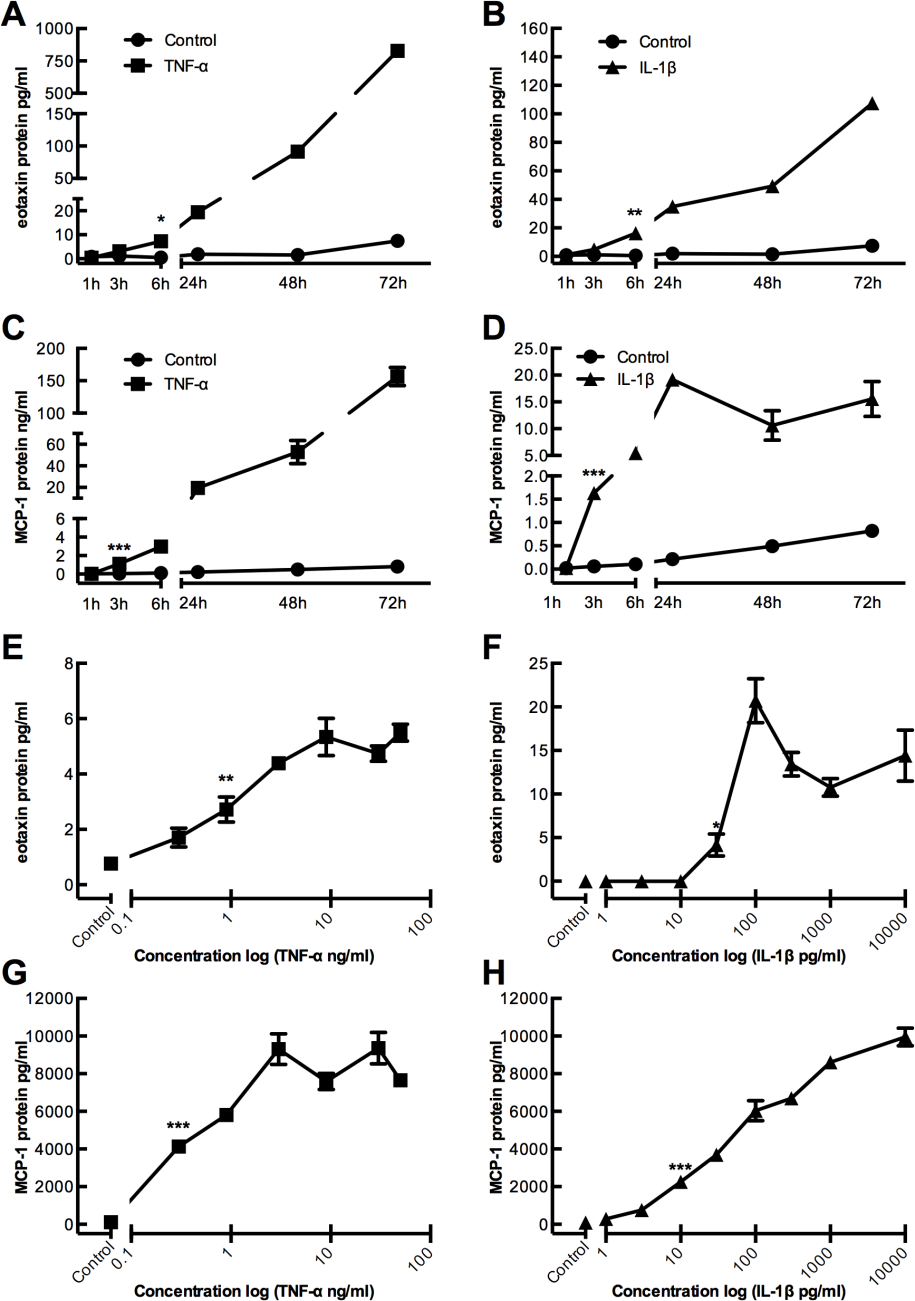
Time- and dose-dependent increase of eotaxin and MCP-1 protein expression in gingival fibroblasts stimulated by TNF-α and IL-1β. **(A and C)** TNF-α (50 ng/ml) stimulates eotaxin and MCP-1 protein expression, and **(B and D)** IL-1β (100 pg/ml) stimulates eotaxin and MCP-1 protein expression a time-dependent manner. **(E and G)** TNF-α stimulates eotaxin and MCP-1 protein expression, and **(F and H)** IL-1β stimulates eotaxin and MCP-1 protein expression in a dose-dependent manner.

Given that gingival fibroblasts are the most abundant cell in gingival connective tissue they are potential contributors to chemokine production in periodontitis. We therefore assessed if isolated gingival fibroblasts constitutively expressed eotaxin and MCP-1 and if their expression was regulated in response to pro-inflammatory stimuli. Human gingival fibroblasts were cultured at increasing time-points (1h, 3h, 6h, 24h, 48h and 72h) in the absence or presence of TNF-α (50 ng/ml) or IL-1β (100 pg/ml). Protein analysis using ELISA showed that the cells constitutively expressed eotaxin and MCP-1 protein. Treatment of the cells with IL-1β or TNF-α resulted in a time-dependent upregulation of eotaxin expression with statistically significant increase at 6h in the presence of either TNF-α (*P*<0.05) (Fig 3A), or IL-1β (*P*<0.01) (Fig 3B). A time-dependent upregulation of MCP-1 protein expression was also seen in cells incubated with TNF-α (Fig 3C) or IL-1β (Fig 3D) with a significant (*P*<0.001) effect observed after 3h with TNF-α or IL-1β exposure.

The protein expression of eotaxin was upregulated by TNF-α in a concentration-dependent manner, with a significant increase detected at 0.9 ng/ml (*P*<0.01) of TNF-α (Fig 3E), and at 30 pg/ml of IL-1β (*P<*0.05) (Fig 3F). The protein expression of MCP-1 was also upregulated in a concentration-dependent manner in response to TNF-α (Fig 3G) and IL-1β (Fig 3H), with significant increase detected at 0,3 ng/ml *P*<0.001) and 10 pg/ml (*P*<0.001), respectively. Thus, human gingival fibroblasts strongly upregulate two leukocyte attracting chemokines in response to pro-inflammatory stimuli.

### Time- and concentration dependent regulation of eotaxin and MCP-1 gene expression by TNF-α and IL-1β in primary human gingival fibroblasts

**Fig 4:**
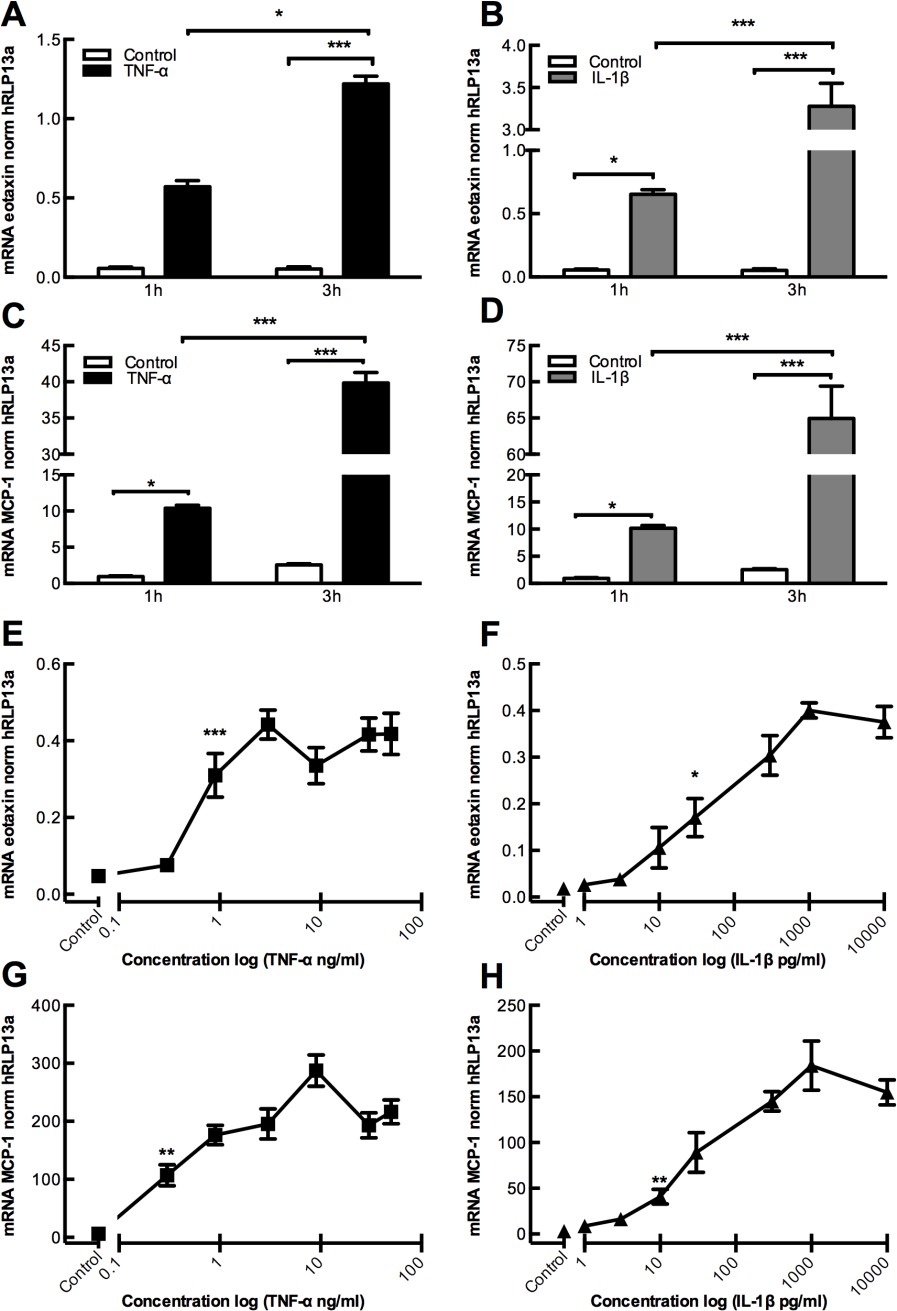
Time- and dose-dependent increase of eotaxin and MCP-1 mRNA expression in gingival fibroblasts by TNF-α and IL-1β. **(A and C)** TNF-α (50 ng/ml) increases eotaxin and MCP-1 mRNA expression, and **(B and D)** IL-1β (100 pg/ml) increases eotaxin and MCP-1 mRNA expression in a time-dependent manner. Analysis performed at 1h and 3h. **(E and G)** TNF-α increases eotaxin and MCP-1 mRNA expression, and **(F and H)** IL-1β increases eotaxin and MCP-1 mRNA expression in a dose-dependent manner. Analysis performed at 24h. Data expressed as means ± SEM. Data represent three individual experiments.

To investigate if the enhanced protein expression of eotaxin and MCP-1 was due to transcriptional regulation we next analyzed mRNA expression of *CCL11* (encoding eotaxin) and *CCL2* (encoding MCP-1) in human gingival fibroblasts in the absence or presence of TNF-α (50 ng/ml) or IL-1β (100 pg/ml). TNF-α and IL-1β gradually enhanced *CCL11* and *CCL2* mRNA expression at 1 and 3h (Fig 4A and 4B). Both cytokines also gradually upregulated *CCL2* mRNA at 1 and 3h (Fig 4C and 4D). TNF-α concentration-dependently upregulated the *CCL11* and *CCL2* mRNA expression (Fig 4E and 4F), at similar concentrations that increased eotaxin and MCP-1 protein expression. Similarly, IL-1β upregulated *CCL11* and *CCL2* mRNA expression (Fig 4G and 4H), at concentrations in the same range as those increasing eotaxin and MCP-1 protein.

### NF-κΒ activation is required for TNF-α and IL-1β stimulated eotaxin and MCP-1 gene expression in gingival fibroblasts

**Fig 5:**
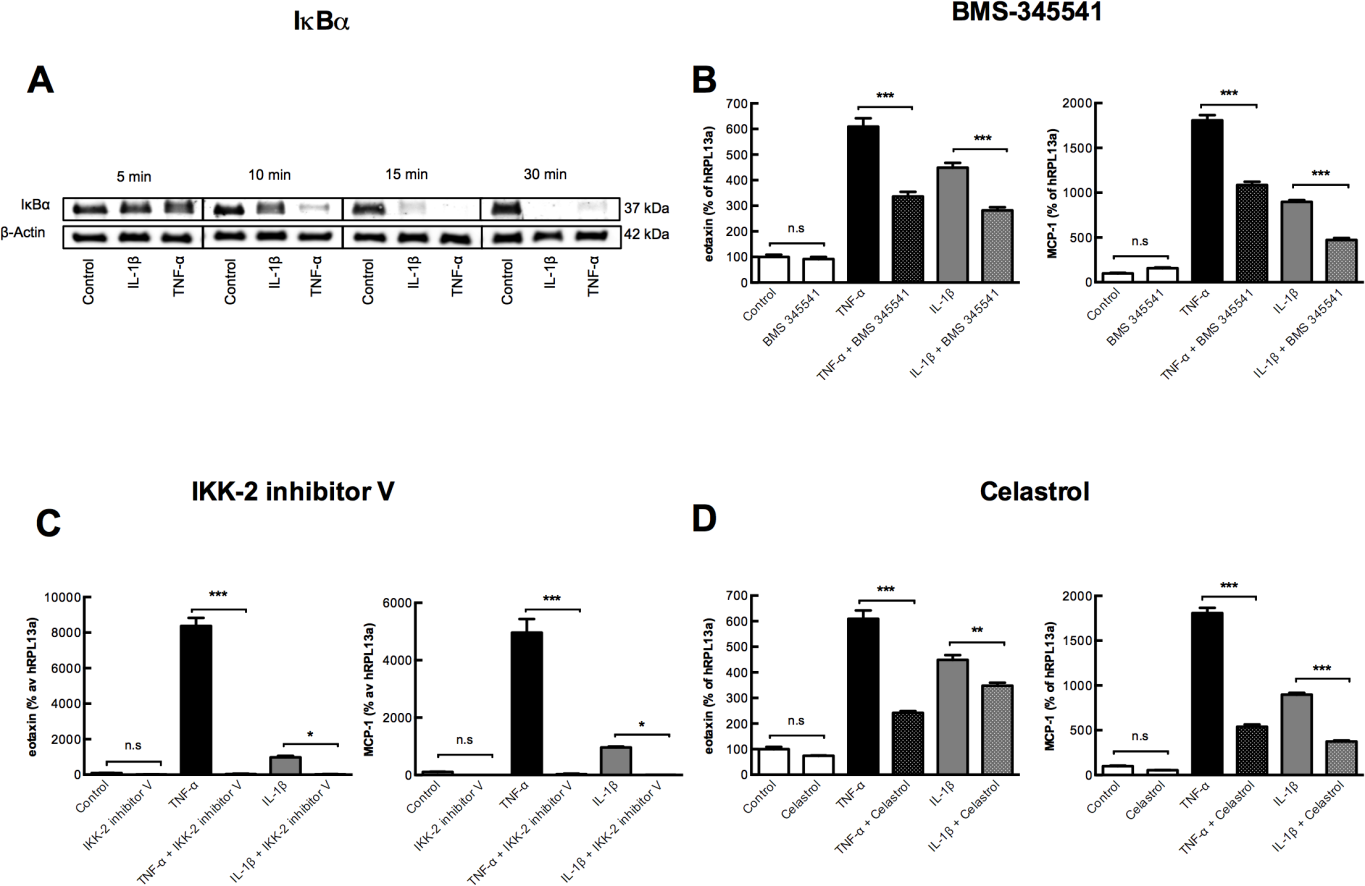
The canonical pathway of NF-κB activation is required for TNF-α and IL-1β stimulated eotaxin and MCP-1 gene expression in gingival fibroblasts. **(A)** Time-dependent decrease of IκBα protein in gingival fibroblasts exposed to TNF-α (50 ng/ml) and IL-1β (100 pg/ml) cultured for 5, 10, 15 and 30 min. The pharmacological inhibitors **(B)** BMS-345541 (6 μM) and **(C)** IKK-2 inhibitor V (10 μM) **(D)** Celastrol (0.1 μM) inhibit the expression of eotaxin and MCP-1 mRNA in gingival fibroblasts exposed to TNF-α (10 ng/ml) and IL-1β (30 pg/ml). Analysis performed at 48 h. Data expressed as means ± SEM.

**Fig 6:**
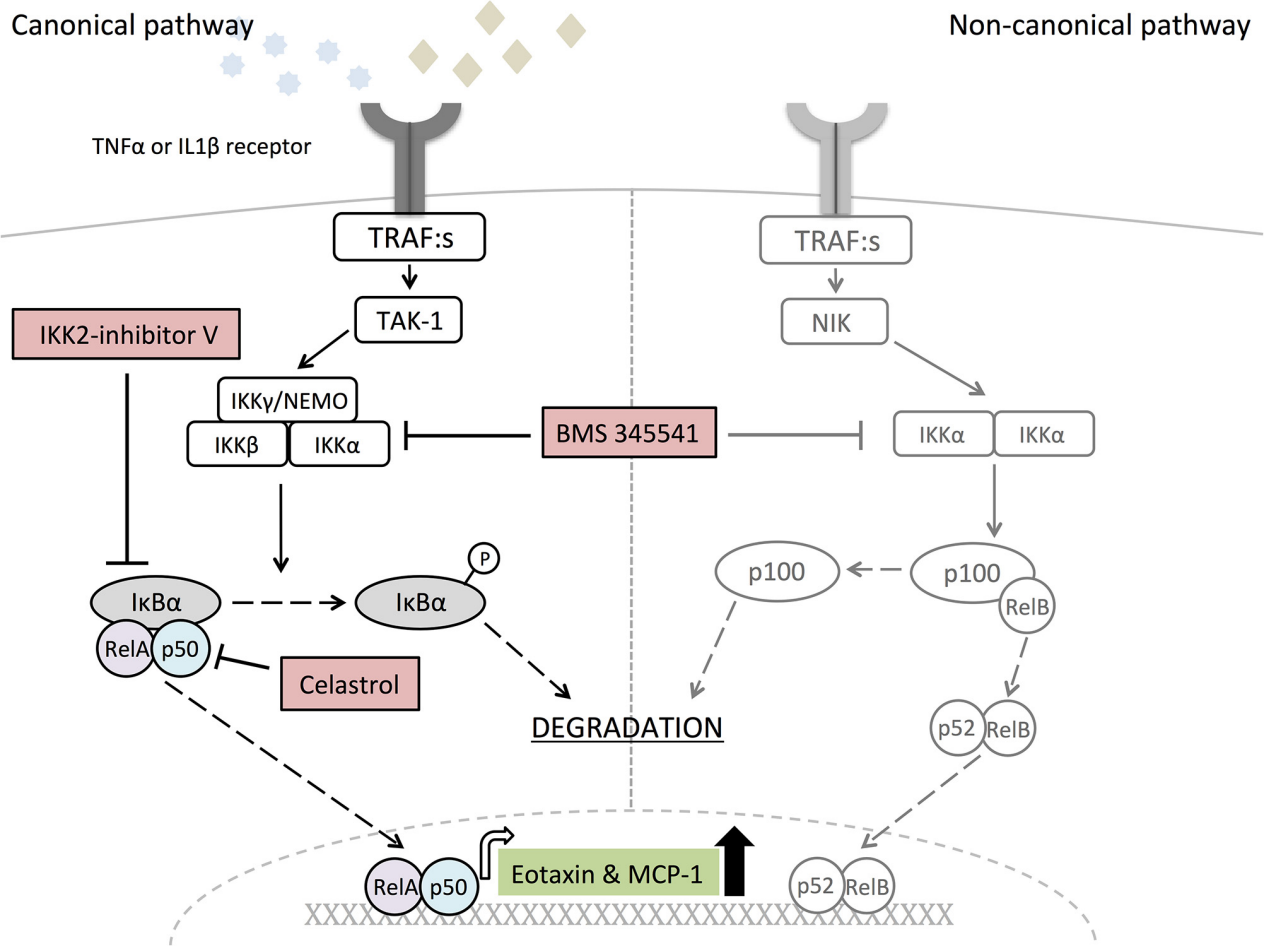
Schematic figure of the canonical versus non-canonical NF-κB signaling pathways, and downstream targets of inhibitors.

We next investigated by which intracellular signaling mechanisms IL-1β and TNF-α upregulate *CCL11* and *CCL2* mRNA expression. Since NF-κB is a well-recognized transcription factor in the pro-inflammatory signaling downstream both the IL-1 and TNF receptors, and is a regulatory element in both the *CCL11* and *CCL2* promoters, we initially evaluated the role of NF-κB in regulation of *CCL11* and *CCL2* mRNA expression in the human gingival fibroblasts. As expected, both IL-1β and TNF-α activated NF-κB in these cells, assessed by decreased amounts of IκBα protein (Fig 5A and S2 Fig). To analyze if the activation of NF-κΒ contributed to the increased expression of *CCL11* and *CCL2* we used three different pharmacological inhibitors of NF-κB. Addition of the IKK inhibitor BMS 344551 (inhibitor of both IKKα and IKKβ) resulted in 30–50% inhibition of TNF-α and IL-1β stimulated *CCL11* and *CCL2* expression (Fig 5B). Addition of a specific IKKβ inhibitor (IKK-2 inhibitor V) completely abolished the *CCL11* and *CCL2* mRNA response to both TNF-α and IL-1β (Fig 5C). Furthermore, addition of Celastrol, an inhibitor of p50 which is downstream of IKKβ activation, resulted in a 30–70% inhibition of TNF-α and IL-1β stimulated *CCL11* and CCL2 expression (Fig 5D). None of the inhibitors affected the basal expression of eotaxin and MCP-1 (Fig 5B–5D). These findings suggest that the increased expression of *CCL11* and *CCL2* in response to the pro-inflammatory cytokines TNF-α and IL-1β is mediated via the canonical pathway of NF-κΒ signaling (Fig 6).

## Discussion

Serologic biomarkers, including auto-antibodies, cytokines, and chemokines, are widely used in the diagnosis, prognosis evaluation, and surveillance of inflammatory and autoimmune diseases [26]. Despite the evolving field of defective systemic host response in periodontitis and the link to atherosclerosis [3] and RA [6], few serologic markers have been identified and little is known about periodontal disease susceptibility. We analyzed a spectrum of cytokines and chemokines in serum from individuals with and without periodontitis and could through multivariate PLS modeling identify eotaxin and MCP-1, CRP, years of smoking and age as periodontitis associated factors. Most interestingly, a preliminary data screening performed by de Quiroz *et al*. demonstrated high eotaxin serum levels in a small Brazilian periodontits cohort [27]. To our knowledge their finding have yet not been replicated in any other study. Two previous studies show increased serum levels of MCP-1 in periodontitis. To our knowledge, we are the first to show the explanatory power of these chemokines in periodontitis using multivariate modeling including known disease confounders, and our results validate and strengthen the association between eotaxin and MCP-1 with periodontitis found by others [27–29].

Chemokines are broadly divided into four sub-families according to their structure and spacing of cysteine residues, namely CXC, CC, C and CX3C. The CXC chemokines enable recruitment of neutrophils and predominantly include IL-8, whereas the CC chemokines are critical to the migration of monocytes and T lymphocytes. Eotaxin and MCP-1 are both CC chemokines partly homologous with 64% shared amino acid identity. Eotaxin is a chemoattractant for different immune cells such as eosinophils, basophils, myeloid cells and T-helper 2 lymphocytes and acts through the CC chemokine receptor 3 (CCR3) [30]. MCP-1 is one of the most potent chemoattractants for monocytes acting through the CC chemokine receptor 2 (CCR2) [31]. There is no previous report on eotaxin expression in periodontal tissues so we measured eotaxin protein in tissue lysates. Surprisingly, despite the found high expression of eotaxin in gingival fibroblasts, we were unable to detect eotaxin in tissue lysates by ELISA. This could be due to rapid section or regulatory mechanisms of eotaxin locally. However, to confirm the presence of eotaxin in gingival tissue we performed immunohistochemistry and could confirm eotaxin expression and localization to gingival fibroblasts (data not shown). Marked expression of MCP-1 has been observed in gingival tissue of adult periodontitis patients [32,33] and we show that the protein levels of MCP-1 are higher, compared to healthy gingiva, suggesting a role in monocyte chemotactic activity in the inflamed gingiva and a possible leakage to serum.

In the present paper, we demonstrate a correlation between MCP-1 serum levels and periodontitis and moreover, a correlation between serum MCP-1 levels and BMI. This is interesting in relation to the fact that overweight and obesity is regarded as a low-grade inflammation state, and that associations to inflammatory diseases such as periodontitis and atherosclerosis have been described. A meta-analysis summarizing results from 28 published studies reported an odds ratio of 1.35 (95% CI: 1.23, 1.47) for the association between periodontitis and obesity [5]. In the present study the association between periodontitis and BMI did not reach statistical significance (*P* = 0,055), possibly explained by too few individuals included in the study. The potential relationship between obesity and periodontitis is biologically complex and poorly understood. Pro-inflammatory cytokines are often elevated in obesity [34] which may contribute to the pathophysiology of periodontitis in obese people [35]. Macrophage infiltration in adipose tissue is common and drives adipocyte differentiation and secretion of pro-inflammatory cytokines such as TNF-α and IL-6 [34]. In this study, we show increased CRP-levels in periodontitis which is in accordance with several previous studies [2]. This finding, together with the finding that periodontal therapy lowers CRP points to a systemic involvement in periodontitis [1]. Previous studies that show increased serum levels of MCP-1 in periodontitis [28,29], do not present MCP-1 data in relation to BMI. It is, therefore, difficult to evaluate the correlation between periodontal inflammation and MCP-1 serum levels in these studies. In obese humans and rodents, MCP-1 is expressed by adipose tissue and increases proportionally to adiposity. Moreover, weight loss causes a decrease in MCP-1 levels, and MCP-1 levels are reduced following periodontal treatment [28]. In obesity, MCP-1 is negatively correlated with high-density lipoprotein levels, and positively with insulin resistance. This indicates that MCP-1 can be a potential candidate linking obesity with metabolic complications, such as atherosclerosis, diabetes, and possibly periodontitis. Interestingly, we show increased CRP and MCP-1 levels in normal weight patients, thus suggesting that both BMI and periodontitis can contribute to a systemic increase in these markers. Eotaxin and its receptor, CCR3, are overexpressed in human atherosclerosis, suggesting an involvement in vascular inflammation [36]. Therefore, our findings of high eotaxin, MCP-1, and CRP systemically in periodontitis patients suggest a potential link to metabolic diseases. Eotaxin could be a potential biomarker for periodontitis but due to the observed association between serum MCP-1 and BMI the validity of MCP-1 as a potential biomarker for periodontitis is questionable.

Cigarette smoking is a major risk factor for periodontal health and there are reports indicating that smoking can influence the expression of inflammatory markers systemically [37, 38]. It is therefore important to take smoking into account when analyzing inflammatory markers in periodontitis. Increased levels of MCP-1 in serum and GCF have previously been observed in smokers with periodontitis compared to non-smoking periodontitis patients and healthy subjects [39, 40] and Souto et al. reported an altered chemokine expression in gingival tissue samples from smokers with chronic periodontitis compared to non-smoking patients [41]. There are no reports about smoking and effects of eotaxin serum- or gingival levels in individuals with periodontitis. We found no difference in the chemokine expression between smokers and never smokers through PCA analysis. However, the prevalence of smoking is low in the study population, which in combination with the comparably limited study cohort may lead to that this evaluation is underpowered. Thus, in order to assess the impact of smoking on MCP-1 and eotaxin levels in periodontitis, studies in larger cohorts are needed.

Fibroblasts are likely to participate in the regulation of immune responses in periodontal tissue by secretion of a variety of cytokines involved in bone and tooth supporting tissue remodeling. We have earlier shown that gingival fibroblasts secrete osteotropic IL-6 type cytokines [42], and the macrophage growth factors M-CSF and IL-34, which are involved in myeloid cell recruitment, survival and osteoclastogenesis [43]. Our findings here that gingival fibroblasts also secrete eotaxin and MCP-1 further support the role of these cells in leukocyte recruitment. Pro-inflammatory cytokines induce MCP-1 in synovial fibroblasts [44] and MCP-1 and eotaxin in dermal fibroblasts [45]. A previous report show induced eotaxin protein in response to TNF-α and IL-1β in gingival fibroblasts [46], and we further demonstrate that eotaxin protein and mRNA expression is time- and dose-dependent in these cells. Our finding that TNF-α and IL-1β induce both MCP-1 protein and mRNA expression is supported by previous studies showing induced MCP-1 mRNA in response to IL-6, IL-1β [47], and *Porphyromonas gingivalis* [48] in gingival fibroblasts.

To be able to monitor and potentially target the expression of eotaxin and MCP-1 in gingival fibroblasts knowledge of the intra cellular signaling pathway is needed. The transcription of several cytokines and chemokines involves NF-κB signaling. In an inactivated stage, NF-κB dimers are bound to the inhibitor IκBα, which retain NF-κB in the cytosol. In the canonical or classical activation pathway, in response to inflammatory mediators such as TNF-α and IL-1β, IκB kinase IKKβ, within the multisubunit IKK complex, phosphorylates IκBα, leading to its ubiquitination and degradation, allowing NF-κB to translocate to the nucleus [49]. We show that TNF-α and IL-1β decrease IκBα protein in a rapid in a time-dependent manner, demonstrating that the canonical pathway is activated. Using three different pharmacological NF-κB inhibitors we are the first to show that the canonical NF-κB signaling mediates pro-inflammatory induced chemokine expression in gingival fibroblasts (schematic, Fig 5E). The transcription of several cytokines involved in RA [50], arthrosclerosis [51], and colitis [52] depend on dysregulated NF-κB signaling. The highly selective and orally bioavailable IKK inhibitor BMS-345541 exhibits anti-inflammatory properties and prevent bone erosions in different animal arthritis models [53]. Moreover, pharmacological inhibitors of IKK inhibit critical signaling pathways that regulate osteoclast formation and survival, and prevent ovariectomy-induced bone loss *in vivo* [54]. In this context, our finding that an IKKβ inhibitor blocks chemokines in gingival fibroblast is interesting as such inhibitors could reduce inflammation. Further studies will however be directed towards modulation of involved chemokines as this approach may be beneficial and have less side effects.

In summary, we identify higher serum levels of CRP, eotaxin and MCP-1 in subjects with periodontitis. This, together with our finding that both CRP and MCP-1 correlates with BMI points towards an increased systemic load of inflammatory mediators in patients with periodontitis and high BMI. We also show expression of eotaxin and MCP-1 in gingival fibroblasts under inflammatory conditions. Further studies are needed to elucidate the prognostic value of eotaxin and MCP-1, as well as modulation of these *in vivo* in experimental periodontitis models.

## Supporting Information

S1 Fig Multivariate PCA modeling of inflammatory mediators in serum from never and present and smokers. The scores t1 and t2 are the new PCS created variables summarizing the x variables. The oval circle illustrates the tolerance ellipse based on Hotelling´s of T2, any observation located outside of the ellipse would be an outlier. Exclusion of outliers did not alter the pattern, i.e. no clustering appeared.
(TIF)

S2 Fig Uncropped and unadjusted western blot data. (JPG)

S1 Table Serum levels of cytokines in subjects with periodontitis versus periodontally healthy. (DOCX)
