# Supplementary figures and images for: Increased Eotaxin and MCP-1 Levels in Serum from Individuals with Periodontitis and in Human Gingival Fibroblasts Exposed to Pro-Inflammatory Cytokines
Source: PLoS One. 2015 Aug 4;10(8):e0134608. doi: 10.1371/journal.pone.0134608 (PMC4524692; doi:10.1371/journal.pone.0134608)

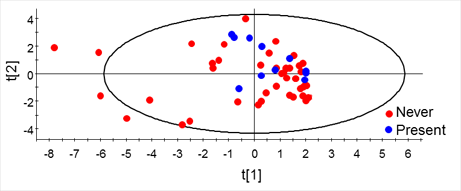

Supplement: S1 Fig — The scores t1 and t2 are the new PCS created variables summarizing the x variables. The oval circle illustrates the tolerance ellipse based on Hotelling´s of T2, any observation located outside of the ellipse would be an outlier. Exclusion of outliers did not alter the pattern, i.e. no clustering appeared. (TIF) [file pone.0134608.s001.tif]

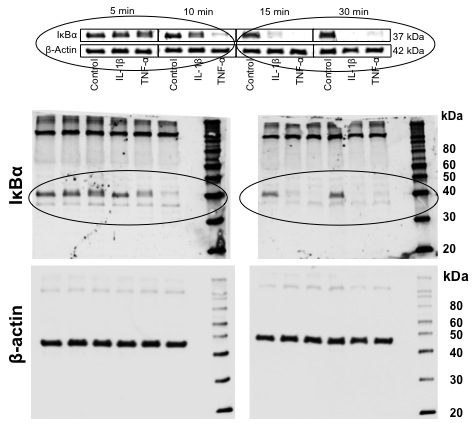

Supplement: S2 Fig — (JPG) [file pone.0134608.s002.jpg]
