# Supplementary material for: Increased Eotaxin and MCP-1 Levels in Serum from Individuals with Periodontitis and in Human Gingival Fibroblasts Exposed to Pro-Inflammatory Cytokines
Source: PLoS One. 2015 Aug 4;10(8):e0134608. doi: 10.1371/journal.pone.0134608 (PMC4524692; doi:10.1371/journal.pone.0134608)
Supplement: S1 Table — (DOCX) [file pone.0134608.s003.docx]

**Supplementary Table 1.** Serum levels of cytokines in subjects with periodontitis (PD) versus periodontally healthy (PH).

|  | Concentration (pg/ml)^a^ | | |  | Per cent with non-detectable concentration^b^ | | |
| --- | --- | --- | --- | --- | --- | --- | --- |
|  | PD | PH | P-value |  | PD | PH | P-value |
| IL-1β | 0.10 (0.10-23.7) | 0.10 (0.10-16.7) | 0.582 |  | 70.0 | 75.0 | 0.617 |
| IL-4 | 0.10 (0.10-76.3) | 0.10 (0.10-49.2) | 0.378 |  | 75.0 | 82.5 | 0.412 |
| IL-6 | 0.10 (0.10-15.5) | 0.10 (0.10-9.8) | 0.382 |  | 75.0 | 82.5 | 0.412 |
| IL-10 | 0.10 (0.10-33.0) | 0.10 0.10 (30.7) | 0.454 |  | 72.5 | 80.0 | 0.431 |
| IL-12 | 0.10 (0.10-39.9) | 0.10 (0.10-69.4) | 0.782 |  | 76.7 | 73.2 | 0.705 |
| IL-13 | 0.10 (0.10-28.9) | 0.10 (0.10-42.6) | 0.679 |  | 77.5 | 87.5 | 0.239 |
| IL-17 | 0.10 (0.10-67.4) | 0.10 (0.10-41.4) | 0.367 |  | 62.5 | 50.0 | 0.260 |
| TNF-α | 0.87 (0.10-11.0) | 1.17 (0.10-12.6) | 0.287 |  | 40.0 | 22.5 | 0.091 |
| IFN-γ | 0.24 (0.10-110) | 0.10 (0.10-200) | 0.921 |  | 50.0 | 57.5 | 0.501 |
| FGF-2 | 0.10 (0.10-146) | 0.10 (0.10-137) | 0.880 |  | 80.0 | 80.0 | 1.000 |
| MIP-1α | 0.10 (0.10-14.8) | 0.10 (0.10-12.7) | 0.820 |  | 80 | 80 | 1.000 |
| MDC | 1346 (672-2609) | 1292 (642-2443) | 0.465 |  | 0 | 0 | 1.000 |

1. Data are presented as medians (5-95% percentile values). Differences between groups were tested with two-sided Mann Whitney U test.
2. Proportions (%) with no or a concentration below the detection level (0.1 pg/ml). Differences in group distributions for having non-detectable versus detectable levels were tested with a two-sided Chi^2^-test.
